# T1 Mapping MOLLI 5(3)3 Acquisition Scheme Yields High Accuracy in 1.5 T Cardiac Magnetic Resonance

**DOI:** 10.3390/diagnostics12112729

**Published:** 2022-11-08

**Authors:** Patrick Krumm, Petros Martirosian, Alexander Brendel, Jens M. Kübler, Jan M. Brendel, Sebastian Gassenmaier, Arne Estler, Meinrad Gawaz, Konstantin Nikolaou, Simon Greulich

**Affiliations:** 1 Department of Radiology, Diagnostic and Interventional Radiology, University of Tübingen, 72076 Tübingen, Germany; 2 Department of Radiology, Section on Experimental Radiology, University of Tübingen, 72076 Tübingen, Germany; 3 Department of Internal Medicine III, Cardiology and Angiology, University of Tübingen, 72076 Tübingen, Germany

**Keywords:** T1 mapping, cardiac magnetic resonance imaging, MOLLI, accuracy

## Abstract

**Objectives:** To systematically compare two modified Look-Locker inversion recovery (MOLLI) T1 mapping sequences and their impact on (1) myocardial T1 values native, (2) post-contrast and (3) extracellular volume (ECV). **Methods:** 200 patients were prospectively included for 1.5 T CMR for work-up of ischemic or non-ischemic cardiomyopathies. To determine native and post-contrast T1 for ECV calculation, two different T1 mapping MOLLI acquisition schemes, 5(3)3 (designed for native scans with long T1) and 4(1)3(1)2 (designed for post-contrast scans with short T1), were acquired in identical mid-ventricular short-axis slices. Both schemes were acquired in native and post-contrast scans. **Results:** Datasets from 163 patients were evaluated (age 55 ± 17 years; 38% female). Myocardial T1 native for 5(3)3 was 1017 ± 42 ms vs. 956 ± 40 ms for 4(1)3(1)2, with mean intraindividual difference −61 ms (*p* < 0.0001). Post-contrast myocardial T1 in patients was similar for both acquisition schemes, with 494 ± 48 ms for 5(3)3 and 490 ± 45 ms for 4(1)3(1)2 and mean intraindividual difference −4 ms. Myocardial ECV for 5(3)3 was 27.6 ± 4% vs. 27 ± 4% for 4(1)3(1)2, with mean difference −0.6 percentage points (*p* < 0.0001). **Conclusions:** The T1 MOLLI 5(3)3 acquisition scheme provides a reliable estimation of myocardial T1 for the clinically relevant range of long and short T1 values native and post-contrast. In contrast, the T1 MOLLI 4(1)3(1)2 acquisition scheme may only be used for post-contrast scans according to its designed purpose.

## 1. Introduction

T1 mapping in cardiac magnetic resonance (CMR) has evolved in the last few years into a powerful tool to estimate subtle and diffuse myocardial damage and is increasingly becoming part of routine clinical protocols [1,2,3,4]. Myocardial damage in cardiomyopathy, myocarditis, systemic diseases such as sarcoidosis and systemic sclerosis could be detected with higher sensitivity and at earlier stages [5,6,7,8,9,10].

Different MR sequences can be applied: modified Look-Locker inversion recovery (MOLLI) and saturation recovery single-shot acquisition (SASHA) result in different T1 values [11,12].

MOLLI acquisition schemes are described with numbers representing consecutive heartbeats with image acquisition and (numbers) in parentheses representing undisturbed signal recovery [4]. A MOLLI scheme described as 5(3)3 indicates image acquisition in five consecutive heartbeats, intermittent undisturbed signal recovery for three heartbeats, followed by image acquisition in three consecutive heartbeats, all within in one breath-hold.

Myocardial extracellular volume (ECV) is estimated using native, post-contrast and blood pool T1 values, as well as hematocrit from a blood sample [11,13].

An approach to measure shorter T1 values post-contrast has been previously described [11], switching from a pre-contrast 5(3)3 MOLLI scheme (designed for long T1 values in native scans) to a post-contrast 4(1)3(1)2 MOLLI scheme (designed for short T1 values post-contrast).

This switch in MOLLI schemes has been implemented in the vendor-specific product-type myocardial tissue characterization mapping protocol. Data are lacking to evaluate the impact of the acquisition schemes on myocardial T1 and ECV in a large patient cohort, and whether this approach of different MOLLI schemes for native and post-contrast might be beneficial in routine clinical practice.

The aim of this study was to systematically investigate the influence and accuracy of MOLLI schemes on native and post-contrast myocardial T1 values and on ECV in a cohort of patients with different myocardial diseases. In addition, accuracy was evaluated in healthy volunteers and in vitro in a phantom model as a reference standard.

## 2. Materials and Methods

### 2.1. CMR In Vitro Imaging

Both T1 MOLLI acquisition schemes were compared with an inversion recovery fast spin-echo (IR-FSE) sequence [14] as a reference standard using a phantom sample array of MnCl_2_ (manganese(II) chloride) with increasing concentrations: 0.05; 0.1; 0.2; 0.3; 0.4 mM. In vitro MOLLI measurements were repeated three times each for accuracy and precision evaluation. MOLLI acquisition parameters in vitro were identical to those for in vivo imaging. Sequence parameters are shown in the Supplementary File.

### 2.2. CMR Image Analysis In Vitro

Image analysis was performed by an experienced MR physicist and clinical radiologists. T1 values and maps were evaluated from experimental data using a custom-made fitting program in MATLAB R2021b (Mathworks, Natick, MA, USA) on a dedicated workstation and repeatedly measured with the same software as for in vivo maps (cmr42 Version 5.11, Circle Cardiovascular Imaging).

### 2.3. Patient Population

Two hundred patients were enrolled in this prospective, single-center, investigator-initiated trial. Patients who underwent clinically indicated CMR from January 2018 to June 2020 were prospectively recruited. A control group of healthy volunteers (*n* = 12) was investigated with native mapping CMR to derive normal values. The institutional review board approved the study protocol (541/2015BO2) and all participants gave written informed consent.

### 2.4. CMR Image Acquisition in Patients

CMR examinations were performed on a 1.5 T scanner (MAGNETOM Aera, SIEMENS Healthcare, Erlangen, Germany). An 18-channel body matrix coil and 12 channels of the spine matrix coil were used for signal reception. All images were acquired in breath-hold and with ECG triggering.

Both T1 mapping measurements were performed in an identical and consistent exemplary mid-ventricular short-axis slice.

Two T1 mapping MOLLI sequences (MyoMaps, SIEMENS Healthcare) with acquisition schemes 5(3)3 (vendor label T1 long) and 4(1)3(1)2 (vendor label T1 short) were acquired consecutively, one immediately after the other, with breath commands in between, in a total acquisition time of maximum 60 s for both MOLLI schemes. Both T1 mapping schemes were acquired in native and 15–20 min post-contrast scans (gadobutrol, (Gadovist^®^, Bayer Healthcare), with a dosage of 0.15 mmol/kg body weight).

To control potential confounders, a comprehensive CMR protocol with T2 and T2* mapping (to exclude edema and iron overload) [15,16] was performed. For T2 mapping, a T2 prepared steady-state free precession (SSFP) sequence with preparation times of 0, 24 and 55 ms was used (MyoMaps, SIEMENS Healthcare). For T2* mapping, a multi-echo gradient echo sequence was used (MyoMaps, SIEMENS Healthcare).

Parameters of all sequences are provided in detail in the Supplementary File.

### 2.5. CMR Image Analysis in Patients

Image analysis was performed by two readers in consensus at an offline workstation using dedicated software (cmr42 Version 5.11, Circle Cardiovascular Imaging). T1 and T2 mapping native, post-contrast and ECV were evaluated as recommended by current SCMR guidelines [4,17]. Myocardial T1, T2 and ECV maps were calculated for the entire mid-ventricular slice. Endomyocardial contours were drawn manually. To prevent partial volume effects, an offset of 15% from manually drawn contours was chosen. Inter-reader variability was assessed in 20 patients. Myocardial T2* mapping was evaluated in an artifact-free region of interest (ROI) in the myocardial septum.

### 2.6. Statistical Analysis

Statistical analysis was performed with JMP (Version 15.1.0, SAS Institute Inc.) and SPSS 26.0.0.1 (IBM Corp.). Data are presented as frequency n (%); mean ± SD (normally distributed); or median [IQR] (not normally distributed), as appropriate. Normality of data was checked visually in data plot curves. Data were compared with the following tests: continuous, normally distributed data were tested with a two-sided *t*-test.

A sub-analysis of T1 and ECV of the three cohorts was performed: intraindividual differences in T1 native vs. post-contrast as well as intraindividual differences in native T1 in 5(3)3 vs. 4(1)3(1)2 MOLLI schemes were compared using analysis of variance (ANOVA).

To investigate the influence of potentially dominating factors, Pearson correlation in normally distributed data for ECV with myocardial and blood pool T1 values native and post-contrast, myocardial T2 and hematocrit was performed. Repetitive measurements for reproducibility were compared with Bland–Altman analysis. The global level of significance *α* was set to 5%. Arithmetical influences of the parameters on ECV within the formula [18] were simulated with standard values and ±50 ms in T1 values and ±5% hematocrit differences.

## 3. Results

### 3.1. CMR Results In Vitro and Simulation

T1 values for MnCl_2_ 0.05 mM were 1467 ms IR-FSE; 1439 ms 5(3)3; 1354 ms 4(1)3(1)2. T1 values for MnCl_2_ 0.3 mM were 431 ms IR-FSE; 406 ms 5(3)3; 408 ms 4(1)3(1)2. Repetitive measurements showed the high intra-method reproducibility of results in a Bland–Altman analysis:

5(3)3: mean difference 0.05% [95%CI limits of agreement −0.6 to 0.7];
4(1)3(1)2: mean difference 0.09% [95%CI limits of agreement −0.3 to 0.5].

**Figure 1:**
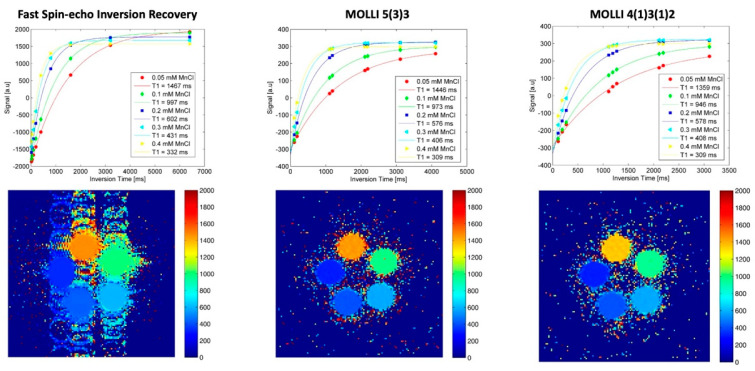
In vitro T1 mapping. As a standard of reference for different T1 relaxation times in the clinically relevant range, a phantom sample array with different concentrations of MnCl_2_ was evaluated with an inversion recovery fast spin-echo (IR-FSE) sequence (left column). T1 evaluation with fitted curves and measured timepoints in the different samples is shown in the top row, with T1 maps including scale in the bottom row. Both MOLLI acquisition schemes (middle and right column) were compared with the same phantom setting.

For detailed T1 mapping results in the phantom, see Table 1 and Figure 1.

Arithmetical influences in factors in the ECV formula are shown in Table 2**.**

### 3.2. CMR Results In Vivo

#### 3.2.1. Study Cohort

Patients with incomplete mapping datasets or impaired image quality (image artefacts) were excluded: *n* = 37. Overall, 163 patient datasets remained evaluable, age 55 ± 17 years, *n* = 62 (38%) were female. Clinical indications for CMR were work-up of ischemic cardiomyopathy (ischemia in coronary artery disease and myocardial infarction, *n* = 42); myocarditis/pericarditis (*n* = 45) or another non-ischemic cardiomyopathy (*n* = 76). Hematocrit value was 40 ± 5% [total range 23; 52%]; blood sample was taken at median 1 day [IQR 0–8; total range 0–31] from CMR.

#### 3.2.2. Native Myocardial Mapping

In patients, myocardial T1 native for 5(3)3 was 1017 ± 42 ms vs. 956 ± 40 ms for 4(1)3(1)2; mean intraindividual difference −61 ms; *p* < 0.0001. In healthy controls, mean intraindividual difference −66 ms (*p* = 0.0002) for native T1 was similar as in patients: myocardial T1 native for 5(3)3 was 991 ± 31 ms vs. 925 ± 24 ms for 4(1)3(1)2. Myocardial T2 was 48 ± 3 in patients. Myocardial iron overload was excluded in all patients, with mean T2* 35 ± 5 ms.

#### 3.2.3. Post-Contrast T1 and ECV Mapping

In patients, post-contrast myocardial T1 was similar for both acquisition schemes with 5(3)3, it was 494 ± 48 ms vs. 490 ± 45 ms for 4(1)3(1)2; mean intraindividual difference −4 ms. Myocardial ECV for 5(3)3 was 27.6 ± 4% vs. 27 ± 4% for 4(1)3(1)2; mean difference −0.6 percentage points; *p* < 0.0001. Myocardial ECV calculated with MOLLI scheme switch 5(3)3 for native scan and 4(1)3(1)2 for post-contrast scan was 28.2 ± 4%; mean difference compared to only 5(3)3 native and post-contrast scan 0.6 percentage points; *p* = 0.0003.

**Figure 2:**
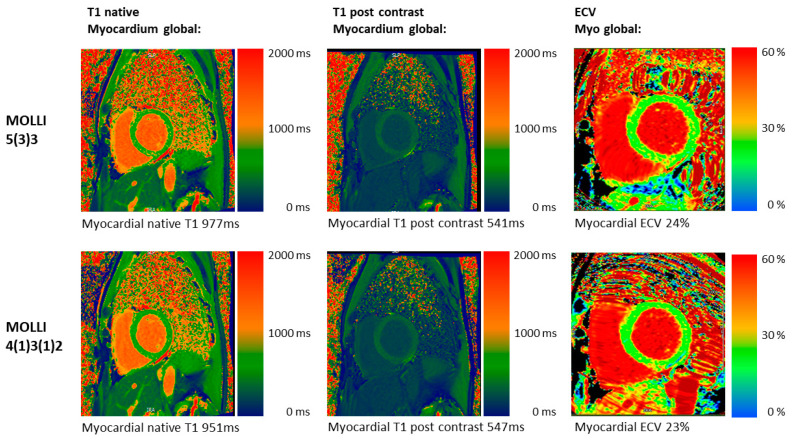
Clinical T1 mapping images in vivo. CMR images of a 48-year-old male patient with an athlete’s heart and screening for DCM with both MOLLI acquisition schemes: 5(3)3 (top row) and 4(1)3(1)2 (bottom row). The native T1 images show lower native T1 values for 4(1)3(1)2 (blue colors) compared to 5(3)3. Post-contrast and ECV are similar for both acquisition schemes.

**Figure 3:**
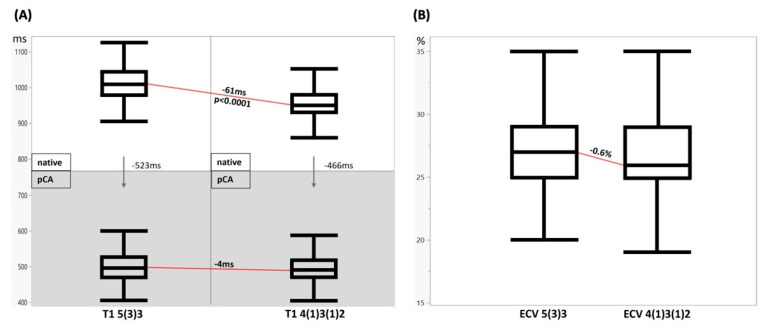
In vivo T1 mapping and ECV calculation results. (**A**) Myocardial T1 native vs. post-contrast agent 5(3)3 vs. 4(1)3(1)2. Myocardial T1 native (top row) vs. post-contrast agent (bottom row, grey) for both MOLLI sequence types, 5(3)3 (left) vs. 4(1)3(1)2 (right). The arrows visualize the signal drop caused by contrast agent application. The red lines show the systematic underestimation of native myocardial T1 and almost no intraindividual difference post-contrast in 4(1)3(1)2 MOLLI. (**B**) Myocardial ECV 5(3)3 vs. 4(1)3(1)2. ECV calculation is similar with both methods. Myocardial ECV estimation was systematically slightly lower for 4(1)3(1)2 sequence.

For clinical images, see Figure 2. For detailed T1 mapping values in patients, see Table 3 and Table 4, and Figure 3A,B.

#### 3.2.4. Sub-Analysis of T1 and ECV by Diagnosis

Sub-analysis of T1 and ECV of the three cohorts revealed higher native T1 for ischemic (1007 ms) and non-ischemic (1012 ms) cardiomyopathy compared to myocarditis (1037 ms) for the 5(3)3 MOLLI scheme. Correspondingly, the intraindividual T1 difference for 5(3)3 vs. 4(1)3(1)2 was higher in myocarditis (73 ms) compared to ischemic (58 ms) and non-ischemic (57 ms) cardiomyopathy, *p* = 0.02.

The intraindividual drop in T1 from native to post-contrast scan did not differ significantly between groups within each MOLLI scheme:

5(3)3: ischemic 521 ms, non-ischemic 515 ms and myocarditis 540 ms, *p* = 0.1;
4(1)3(1)2: ischemic 465 ms, non-ischemic 462 ms and myocarditis 475 ms, *p* = 0.5.

For detailed data of the sub-analysis, see Table 4.

#### 3.2.5. Inter-Reader Reproducibility

Inter-reader variability in a Bland–Altman analysis was as follows:

5(3)3 native: mean difference 1.9 ms [±1.96 SD limits of agreement −21 to 24.7];
5(3)3 post-contrast: mean difference −8.2 ms [±1.96 SD limits of agreement −32.7 to 16.3];
4(1)3(1)2 native: mean difference −4.5 ms [±1.96 SD limits of agreement −31 to 22];
4(1)3(1)2 post-contrast: mean difference −6.2 ms [±1.96 SD limits of agreement −20.8 to 8.3].

#### 3.2.6. Correlation of Myocardial ECV with Formula Factors (Native and Post-Contrast T1, Hematocrit)

To rule out a dominating factor influencing ECV estimation, the formula factors were correlated.

Correlations for 5(3)3 MOLLI ECV values with the formula factors were weak: myocardial T1 native r = 0.3; myocardial T1 post-contrast r = 0.01; blood pool T1 native r = 0.1; blood pool T1 post-contrast 0.1; hematocrit r = 0.2.

Correlations for 4(1)3(1)2 MOLLI ECV values with the formula factors were weak: myocardial T1 native r = 0.2; myocardial T1 post-contrast r = 0.01; blood pool T1 native r = 0.1; blood pool T1 post-contrast 0.1; hematocrit r = 0.3.

## 4. Discussion

This study systematically evaluates the impact and accuracy of two different MOLLI readout schemes on native myocardial T1 values, post-contrast T1 values and on ECV in both a cohort of patients and healthy volunteers and a phantom model.

### 4.1. Influence on T1 Mapping

Analysis of phantom results revealed high accuracy for T1 estimation by 5(3)3 MOLLI, with only slight underestimation of the ‘true’ T1 values measured with time-intensive reference standard measurements.

In contrast, high T1 values around 1000 ms and above were systematically underestimated by approximately 65 ms with 4(1)3(1)2 MOLLI in vitro. The effect of underestimation decreased for shorter T1 values around 500 ms, which are within the range of post-contrast values.

Sub-analysis of T1 mapping between the cohorts revealed higher native T1 values in myocarditis patients, which significantly increased the intraindividual difference in T1 in 5(3)3 vs. 4(1)3(1)2 by 15 ms, *p* = 0.02. This effect reproduces the increased underestimation of T1 in the higher value range by the 4(1)3(1)2 MOLLI scheme, as demonstrated by the phantom model.

However, T1 mapping is prone to other potential confounders, which we tried to avoid (partial volume, cardiac motion, breath-holding). Nevertheless, some others remain unavoidable: Look-Locker correction, preparation pulse factors and multi-shot SSFP, field strength, signal-to-noise, blood flow and magnetization transfer [19]. Signal variations in MOLLI data are well known and can be explained by the incomplete recovery of longitudinal magnetization between successive signal excitations. In particular, this effect occurs when the time delay between excitations is shorter than the T1 relaxation values. The time delay of one heartbeat in the 4(1)3(1)2 scheme is significantly shorter than in the 5(3)3 scheme (three heartbeats) and therefore contributes more to the signal variation. 

### 4.2. Influence on ECV Calculation Mapping

The effect of the native T1 underestimation of 4(1)3(1)2 MOLLI on myocardial ECV calculation in absolute numbers is only marginal, but is systematic and, hence, statistically significant. This reconfirms the purpose of ECV to equalize differences in acquisition methods and results in similar ECV values.

Since intraindividual differences between both MOLLI schemes post-contrast are only 4 ms, the shift to the 4(1)3(1)2 MOLLI scheme does not seem necessary for post-contrast T1 mapping. The 5(3)3 MOLLI scheme provides a reliable estimation of myocardial T1 for the clinically relevant range of long and short T1 values native and post-contrast.

Given the systematic differences in both methods applied in this study, other confounders of ECV seem rather unlikely. For both MOLLIs, we found only weak correlations of ECV with T1 values of myocardium and blood pool native and post-contrast, as well as myocardial T2 and hematocrit.

The low correlations of ECV with native T1 and other factors in its formula classify ECV as an independent parameter of myocardial morphology.

### 4.3. Comparison to Previous Results

Roujol et al. [11] investigated the same MOLLI schemes on a 1.5 T GE scanner: pre-contrast only 5(3)3 MOLLI and post-contrast only 4(1)3(1)2 MOLLI. In vitro, they found bias at approximately 1000 ms of 26 ms and for 5(3)3 MOLLI, and 28 ms at approximately 600 ms compared to the spin-echo standard, respectively. These results could be confirmed by our phantom study.

### 4.4. Limitations

As a limitation of this study, accuracy evaluation with repetitive measurements was performed in vitro only. In patients, the study was not designed to consider the reproducibility of the measurements [20]. The strength of this study is the measurement of two different T1 MOLLI sequences in identical slices. Signal variation in MOLLI data could, in principle, be considered in T1 calculation but was not considered in this work due to its complexity in the clinical context.

A significant number of clinical datasets (19%) had to be excluded in this study due to the strict inclusion criteria, with the enrolment of only high-quality images.

## 5. Conclusions

The T1 MOLLI 5(3)3 acquisition scheme provides a reliable estimation of myocardial T1 for the clinically relevant range of long and short T1 values pre- and post-contrast, both in vitro and in vivo. Since T1 mapping is an emerging tool in clinical routine, the T1 MOLLI 5(3)3 acquisition scheme should be preferred.

A change in MOLLI scheme from 5(3)3 native to 4(1)3(1)2 post-contrast for ECV estimation is possible but does not seem necessary. Despite being significant, the impact of the MOLLI scheme on ECV calculation is only marginal.

The T1 MOLLI 4(1)3(1)2 scheme systematically and relevantly underestimates higher native myocardial T1 values and should not be used for native T1 measurements but may be used for post-contrast scans.

## Figures and Tables

**Table 1:** In vitro T1 mapping results.

Sequence	MnCl_2_ Concentration (mM)				
	0.05	0.1	0.2	0.3	0.4
**Fast Spin-echo Inversion Recovery**: T1 [ms] *Reference standard*	**1467**	**997**	**601**	**431**	**332**
**MOLLI 5(3)3**: T1 [ms] *Difference to reference [ms]*	**1439** *−28*	**972** *−25*	**577** *−24*	**406** *−25*	**310** *−22*
**MOLLI 4(1)3(1)2**: T1 [ms] *Difference to reference [ms]*	**1354** *−113*	**946** *−51*	**575** *−26*	**408** *−23*	307 *−25*

T1 values of aqueous MnCl_2_ solutions in phantom sample array measured using the reference standard fast spin-echo inversion recovery (IR-FSE) compared to both MOLLI acquisition schemes. All three sequence types show good concordance (green values) over high, mid-range and low T1 values within the clinically relevant range, with only slight underestimation. High T1 values were systematically and relevantly underestimated (red values) by the 4(1)3(1)2 MOLLI acquisition scheme. MnCl_2_: manganese(II) chloride, MOLLI: modified Look-Locker inversion recovery.

**Table 2:** Influencing calculational factors in ECV formula.

Factor	Change	Influence on ECV (27%)
T1 myocardium native (1000 ms)	+50 ms	ECV +1% ↑
T1 myocardium native (1000 ms)	−50 ms	ECV −1% ↓
T1 myocardium post contrast (500 ms)	+50 ms	ECV −5% ↓↓↓
T1 myocardium post contrast (500 ms)	−50 ms	ECV +6% ↑↑↑
T1 blood pool native (1600 ms)	+50 ms	ECV 0% 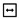
T1 blood pool native (1600 ms)	−50 ms	ECV 0% 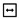
T1 blood pool post contrast (350 ms)	+50 ms	ECV +5% ↑↑↑
T1 blood pool post contrast (350 ms)	−50 ms	ECV −5% ↓↓↓
Haematocrit (41%)	+5%	ECV −2% ↓
Haematocrit (41%)	−5%	ECV +2% ↑

ECV formula: $ECV=\left(1-haematocrit\right)\frac{\frac{1}{post\;contrast\;T1\;myo}-\frac{1}{native\;T1\;myo}}{\frac{1}{post\;contrast\;T1\;blood}-\frac{1}{native\;T1\;blood}}$. Calculated deviation from simulated normal ECV of 27% for all changed parameters in the ECV formula is indicated as absolute percentage point deviation and with arrows: no change 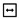, increase **↑**, strong increase **↑↑↑**, decrease **↓**, strong decrease **↓↓↓**. Standard parameters were T1 myocardium native: 1000 ms, T1 myocardium post-contrast: 500 ms, T1 blood pool native: 1600 ms, T1 blood pool post-contrast 350 ms, hematocrit: 41%, ECV: 27%. ECV: extracellular volume.

**Table 3:** T1 mapping in all patients.

n = 163 Patients	T1 5(3)3 MOLLI	T1 4(1)3(1)2 MOLLI	Intraindividual ∆T1 5(3)3 – 4(1)3(1)2
*Myocardium*			
T1 native [ms]	1017 ± 42	956 ± 40	61 ± 32
T1 post CA [ms]	494 ± 48	490 ± 45	4 ± 14
Intraindividual ∆ T1 native-post CA [ms]	523 ± 62	466 ± 59	
ECV [%]	28 ± 4	27 ± 4	1 ± 2
*Bloodpool*			
T1 native [ms]	1581 ± 82	1494 ± 90	87 ± 50
T1 post CA [ms]	348 ± 55	348 ± 55	−1 ± 7

Values are given as mean ± SD; ∆ delta (intraindividual difference). CA: contrast agent, ECV: extracellular volume, MOLLI: modified Look-Locker inversion recovery.

**Table 4:** T1 mapping in patients by diagnosis.

n = 163 Patients	Ischemic Cardiomyopathy (n = 42)		Myocarditis (n = 45)		Non-Ischemic Cardiomyopathy (n = 76)	
	5(3)3	4(1)3(1)2	5(3)3	4(1)3(1)2	5(3)3	4(1)3(1)2
*Myocardium*						
T1 native [ms]	1007 ± 33	948 ± 36	1037 ± 44	964 ± 44	1012 ± 42	955 ± 39
T1 post CA [ms]	486 ± 47	484 ± 43	497 ± 46	489 ± 43	496 ± 49	493 ± 48
ECV [%]	27 ± 3	26 ± 3	28 ± 4	27 ± 5	28 ± 5	27 ± 5
Intraindividual ∆ T1 native-post CA [ms]	521 ± 9	465 ± 9	540 ± 9	475 ± 9	515 ± 7	462 ± 7
Intraindividual ∆T1 5(3)3 – 4(1)3(1)2 [ms]	58 ± 5		73 ± 5		57 ± 3	
*Bloodpool*						
T1 native [ms]	1573 ± 69	1484 ± 83	1596 ± 98	1500 ± 100	1577 ± 77	1496 ± 88
T1 post CA [ms]	338 ± 53	340 ± 55	345 ± 51	345 ± 51	354 ± 59	355 ± 58

Values are given as mean ± SD; ∆ delta (intraindividual difference). CA: contrast agent, ECV: extracellular volume, MOLLI: modified Look-Locker inversion recovery.

## Data Availability

The datasets generated during and/or analyzed during the study are available from the corresponding author on reasonable request.

## Supplementary Materials

The following supporting information can be downloaded at: https://www.mdpi.com/article/10.3390/diagnostics12112729/s1. CMR image acquisition: detailed image parameters.
