# Supplementary material for: T1 Mapping MOLLI 5(3)3 Acquisition Scheme Yields High Accuracy in 1.5 T Cardiac Magnetic Resonance
Source: Diagnostics (Basel). 2022 Nov 8;12(11):2729. doi: 10.3390/diagnostics12112729 (PMC9689660; doi:10.3390/diagnostics12112729)
Supplement: Supplementary file 1 [file diagnostics-12-02729-s001.zip › diagnostics-1964115-supplementary.pdf]

Krumm et al.: T1 Mapping MOLLI 5(3)3 Acquisition Scheme Yields High Accuracy in 1.5 T Cardiac Magnetic Resonance

Supplementary File

CMR image acquisition: Detailed Image Parameters

T1 mapping

Two T1 mapping MOLLI sequences (MyoMaps, SIEMENS Healthcare) with acquisition schemes 5(3)3 (vendor label T1 long); and 4(1)3(1)2 (vendor label T1 short) were acquired consecutively one immediately after the other with breath commands in between. Both T1 mapping schemes were acquired native and 15-20min post contrast after Late Gadolinium Enhancement imaging (LGE).

Sequence Type Steady State Free Precession (SSFP)

5(3)3 (vendor label T1 long) MOLLI:

For RR-interval >700ms: TR 280.6ms, TE 1.12ms, flip angle 35°, slice thickness 8.0mm, baseline matrix 169 x 256.

For RR-interval <700ms: TR 360.6 ms, TE 1.12 ms, flip angle 35°, slice thickness 8.0mm, baseline matrix 169 x 256.

4(1)3(1)2 (vendor label T1 short) MOLLI:

For RR-interval >700ms: TR 263.9ms, TE 1.01ms, flip angle 35°, slice thickness 8.0mm, baseline matrix 169 x 256.

For RR-interval <700ms:

TR 341.4ms, TE 1.01ms, flip angle 35°, slice thickness 8.0 mm, baseline matrix 169 x 256.

Inversion recovery fast spin echo (IR-FSE) in vitro imaging: TR 5000ms, echo time TE 8.2ms; magnetization preparation: slice selective inversion pulse, twelve repeated measurements with inversion time (TI) 25, 30, 35, 40, 60, 100, 200, 400, 800, 1600, 3200, 4800ms; FOV 320x265, matrix 256x159 pixels, slice thickness 5mm, voxel size 1.25x1.67x5mm, GRAPPA factor 2, receiver bandwidth 238 Hz/Pixel.

T2 mapping

For T2 mapping T2 prepared Steady State Free Precession (SSFP) sequence with T2 –preparation pulses (T2prep) at 1) none; 2) 24ms; 3) 55ms was used (MyoMaps, SIEMENS Healthcare).

TR 307.5ms, TE 1.17ms, flip angle 20°, slice thickness 8.0mm, baseline matrix 144 x 192.

T2* mapping

For T2* mapping a multi-echo gradient echo sequence (MyoMaps, SIEMENS Healthcare) with eight echo times (TE) was used.

TR 200ms, TE multiple echo times (2,4-20ms), flip angle 20°, slice thickness 10.0mm, baseline matrix 154 x 256.
